# Attack on Titan (AoT): Anime image dataset for character, scene, emotion recognition and beyond

**DOI:** 10.1016/j.dib.2025.112246

**Published:** 2025-11-08

**Authors:** Guldasta Imraj Khan, Hafeez Anwar, Qasim Jan, Shahid Hussain

**Affiliations:** a Department of Computer Science, National University of Computer and Emerging Sciences (NUCES-FAST, Phase 1 Hayatabad, Peshawar KPK Pakistan; b Department of Business & Accounting, Atlantic Technological University, Ireland

**Keywords:** Computer vision, Machine learning, Image classification, Digital humanities

## Abstract

Anime is an influential medium with global popularity, combining visual aesthetics with narrative depth and offering potential applications in content analysis, style transfer, and emotion recognition within computer vision research. Despite its widespread appeal, publicly available anime character datasets remain scarce. To address this gap, we propose the Attack on Titan: Anime Image Dataset, derived from the popular series *Attack on Titan*, to support anime-focused computer vision research. The dataset comprises 4041 high-quality images divided into 14 classes, each representing a prominent character from the series. These images are manually collected through high-resolution screenshots, capturing a wide range of character poses, expressions, costumes, and backgrounds. The dataset is suitable for various computer vision tasks, including character recognition, emotion detection, style classification, and domain adaptation.

Specifications Table

**Table:** 

Subject	Computer Sciences
Specific subject area	Computer Vision, Deep Learning, Anime Character Recognition
Type of data	Image
Data collection	Dataset is acquired from the videos of the anime series Attack on Titan
Data source location	FAST-NUCES, Peshawar, Pakistan
Data accessibility	Repository name: AOT Characters Dataset Data identification number: 10.17632/fzrj2xc9rt.1 Direct URL to data: https://data.mendeley.com/datasets/fzrj2xc9rt/1
Related research article	*A Challenging Benchmark of Anime Style Recognition* [1]

## 1 Value of the Data

• The lack of well-annotated and publicly available anime character datasets has prevented the development of effective deep learning methods for this domain, targeting various problems such as plagiarism detection [2]. Although existing datasets such as Danbooru and AnimeFace provide valuable resources, they remain limited in number and scope, with most focusing primarily on face-only crops. This restricts their usefulness to narrow tasks such as face recognition or style classification. In contrast, the AOT dataset offers a more comprehensive resource with 4041 high-quality, manually curated images across 14 character-specific classes, capturing not just faces but also full-body poses, diverse motions, expressions, costumes, and backgrounds. This richer coverage enables researchers to address a wider range of computer vision tasks including character recognition, motion analysis, emotion detection, re-identification across episodes and seasons, and scene understanding. Consequently, our proposed dataset extends the utility of anime datasets beyond face-centric applications.
• Anime presents unique visual traits such as exaggerated expressions, stylized hair and eyes - that challenge general- purpose models trained on real-world images [[3], [4]]. The AOT dataset therefore provides a valuable benchmark for domain-specific methods, supporting research in multi-label classification, transfer learning, and domain adaptation.
• Anime is a dynamic reflection of Japanese culture and artistic heritage, influencing global audiences through its storytelling and visual artistry [5]. The AOT dataset contributes to the preservation and academic documentation of anime’s visual legacy, supporting cultural research and digital humanities initiatives.
• The AoT dataset can also serve as an educational tool for students and practitioners learning computer vision techniques through engaging and domain-relevant examples. Furthermore, because the images capture entire scenes with the character in context, the dataset could be extended with bounding box annotations to enable tasks such as object detection, scene parsing, and background-foreground separation thus resulting in broadening its utility beyond character recognition.

## 2 Background

**Fig. 1:**
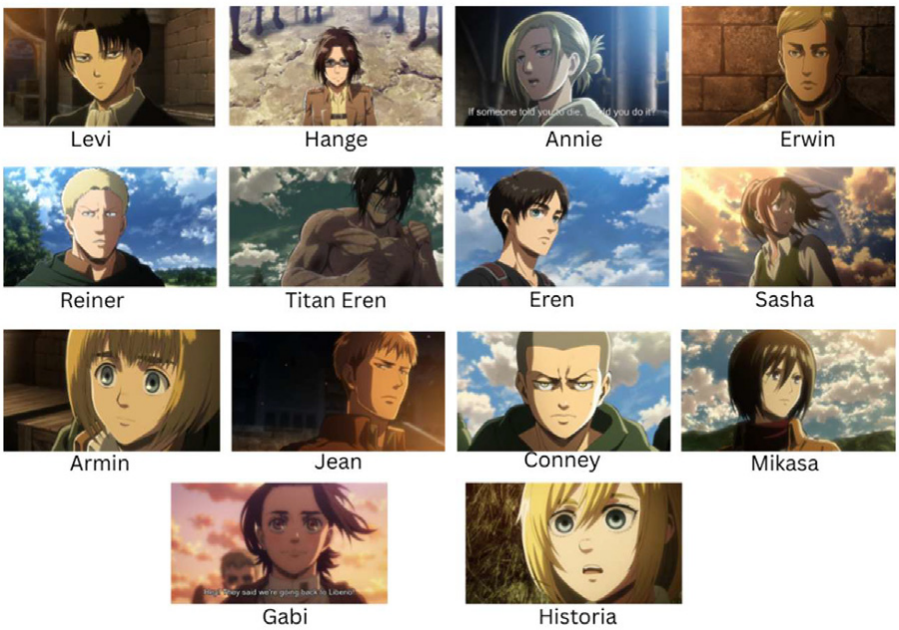
Exemplar images of the anime characters in the dataset.

Anime is a distinctive style of animation that originated in Japan. It is primarily hand-drawn, visually vibrant, and features emotionally expressive characters [6], which include a wide range of themes suitable for all age groups, unlike traditional Western animation [7]. Over the past decade, anime has gained widespread global popularity, with its market valued at USD 33.6 billion in 2024 and projected to reach USD 63.7 billion by 2033 [8]. Some of the most popular and critically acclaimed anime series include Attack on Titan, One Piece, Naruto, and Demon Slayer. As anime continues to gain mainstream recognition, there is increasing interest in developing intelligent systems capable of understanding, classifying, and interacting with anime content [9]. To support this growing interest, clean and well-labelled datasets are essential. Therefore, we introduce the Attack on Titan: Anime Image dataset, featuring characters from the well-known anime series Attack on Titan. The dataset comprises 14 classes, each representing a prominent character, captured across a variety of expressions, poses, and visually diverse scenes. Fig. 1 presents representative samples of the characters included in the dataset, highlighting the diversity in expressions, poses, and visual features. This dataset provides a clean and targeted resource to support researchers in a range of anime-based computer vision tasks, such as character classification, image retrieval, and style analysis. Building upon this foundation, we introduce the Attack on Titan: Anime Image dataset in detail and outline its collection methodology, structure, and potential applications. By making this dataset publicly available to the research community, we aim to accelerate progress in anime-focused computer vision and deep learning and hence contribute to the growing intersection of artificial intelligence and creative media.

## 3 Data Description

**Fig. 2:**
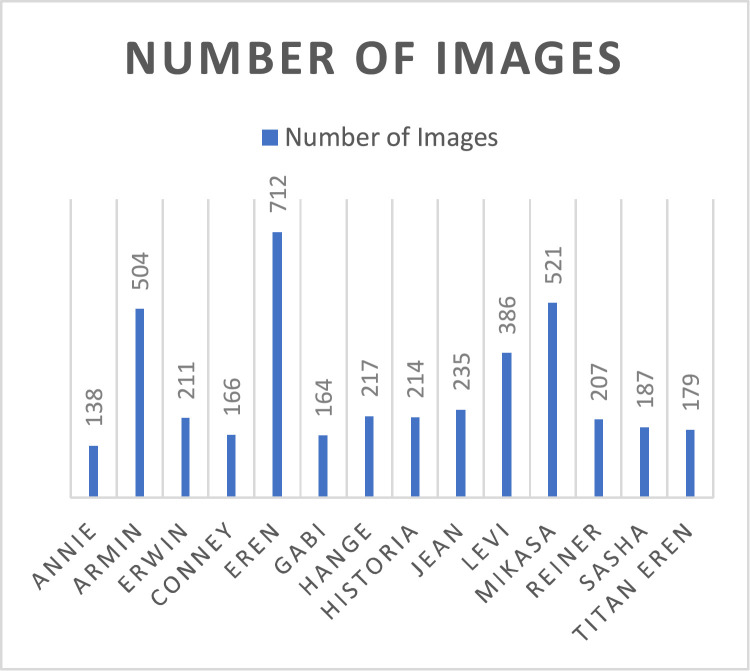
No. of images per character class.

Anime differs from other animated cartoons as it is predominantly hand-drawn and features a highly stylized aesthetic. As a result, anime graphics often include unrealistic elements developed in unique, artistic, or exaggerated ways [6]. Therefore, to ensure consistent and high-quality visual data, careful filtering and deliberate image collection are essential. AOT Characters dataset focuses on 14 prominent characters from the same anime and provides pre-processed, high-resolution images suitable to be used with computer vision and deep learning research. This dataset comprises of 4041 images collected through manual screenshot extraction at an approximate rate of one frame every 10 s, depending on character visibility. Frames where a single target character appeared alone and clearly identifiable were captured, while shots with multiple characters, heavy occlusion, or motion blur were excluded. All images are stored in PNG format with a uniform frame size of 1920×1080 pixels, ensuring visual consistency across classes. The dataset is organized into 14 distinct class folders, each corresponding to a single character, with filenames following a standardized convention(classID_imageID.png). The dataset metadata is summarized in Table 1 and the images frequency per class is shown in Fig. 2.

**Table 1:** Metadata of the AOT dataset showing class IDs, character names, number of images, and file naming conventions.

CLASS ID	CHARACTER NAME	NO. OF IMAGES	FILE NAMING CONVENTION (EXAMPLE)
001	Annie Leonhart	138	001_001.png, 001_002.png, …
002	Armin Arlert	504	002_001.png, 002_002.png, …
003	Erwin Smith	211	003_001.png, 003_002.png, …
004	Conney Springer	166	004_001.png, 004_002.png, …
005	Eren Yeager	712	005_001.png, 005_002.png, …
006	Gabi	164	006_001.png, 006_002.png, …
007	Hange Zoe	217	007_001.png, 007_002.png, …
008	Historia Reiss	214	008_001.png, 008_002.png, …
009	Jean Kirstein	235	009_001.png, 009_002.png, …
010	Levi	386	010_001.png, 010_002.png, …
011	Mikasa Ackerman	521	011_001.png, 011_002.png, …
012	Reiner Braun	207	012_001.png, 012_002.png, …
013	Sasha Braus	187	013_001.png, 013_002.png, …
014	Titan Eren	179	014_001.png, 014_002.png, …

## 4 Experimental Design, Materials and Methods

The images are high-definition screenshots captured using built-in system screenshot functionality across all the four seasons of *Attack on Titan*. These screenshots were taken at an approximate rate of one frame per 10 s, depending on the visibility of the target character throughout all the episodes of the series, covering different scenes with varying lighting conditions, character expressions, and angles. Fourteen key characters are targeted for the dataset based on their importance and screen time in the series. The complete image dataset consists of 14 directories/folders where each directory contains the images depicting each one of the 14-anime characters.

**Fig. 3:**
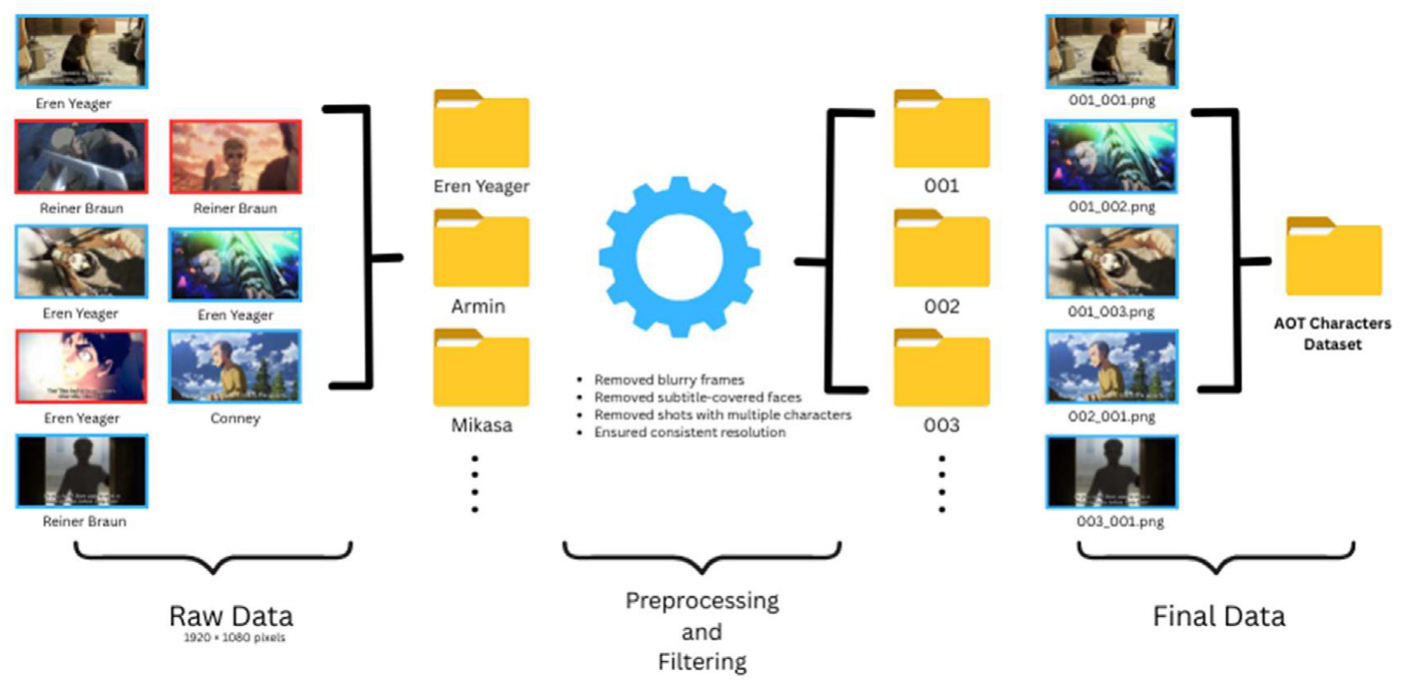
Dataset construction process, showing examples from raw screenshot collection to final refined dataset.

A manual pre-processing step is performed to ensure the quality of the dataset. During this step, all images are individually inspected, and any blurred images (where targeted character was not identifiable even to the human eye) or those with severely occluded discriminative regions - such as faces or key scenes - are removed and. Similarly, frames where the target character appeared alone and clearly identifiable were retained. Image resizing or cropping is not required for the remaining images, as the screenshots were uniformly captured, with all images having the same 1920×1080 pixel resolution. All images are stored in PNG format and preserved in their original size and quality. The whole process is illustrated in Fig. 3. However, it should be noted that this article focuses only on the description of the dataset and hence does not present the elaborate experimental evaluation, training/test splits, and model-specific parameters.

## Limitations

**Fig. 4:**
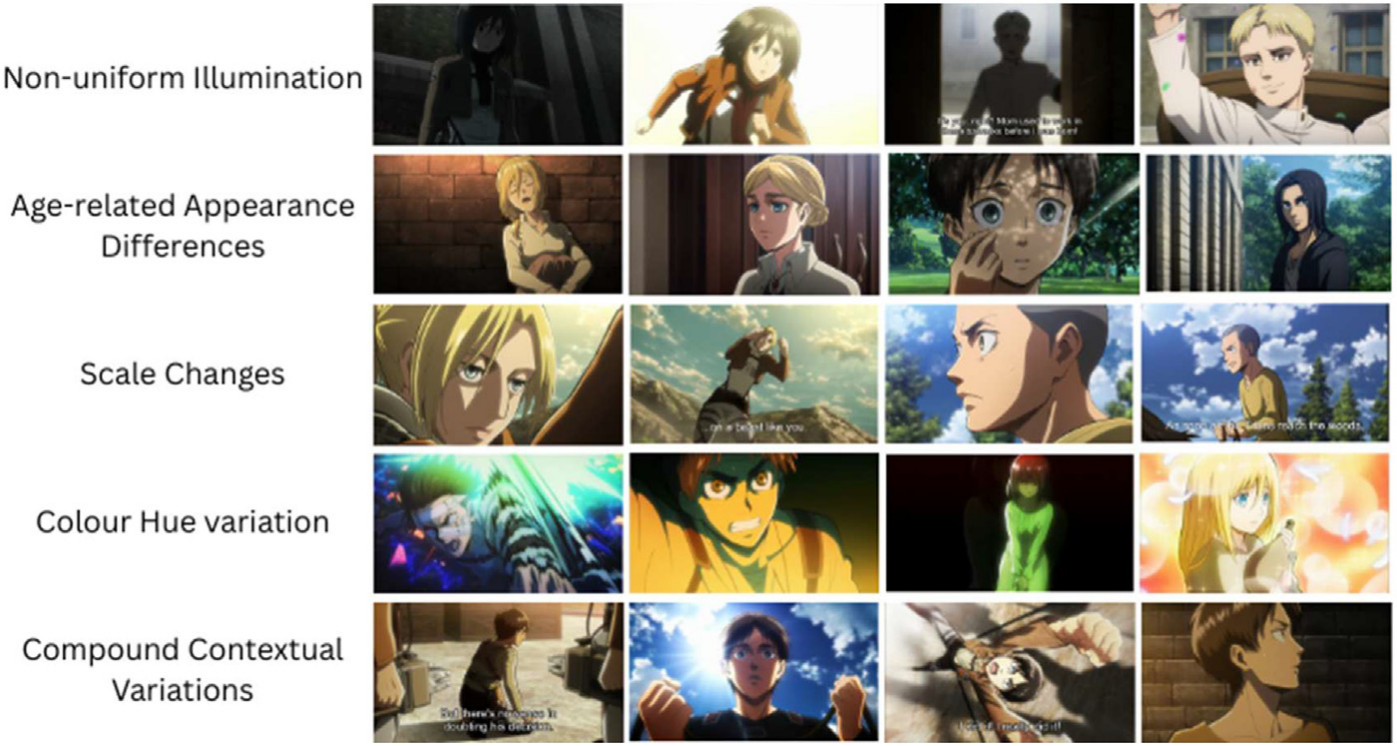
Challenges in the dataset due image variations caused by various factors.
➢ Color hue changes
  As shown in the fourth row of Figure 4, characters even vary in color hue caused by scene-specific tints (e.g., sunset oranges, nighttime blues) and artistic filters introduce non-uniformity in characters’ hair, eyes, and clothing, posing a significant challenge for deep learning algorithms at test time.
➢ Compound contextual variations
  In practice, as shown in the fifth row of Figure 4, multiple factors combine (e.g., a small-scale close-up under colored lighting), amplifying appearance shifts and demanding robust models that can disentangle character identity from background clutter and stylistic embellishments.

Anime character recognition faces several unique challenges due to wide variations in how characters are depicted. These may include:

➢ Non-uniform illumination
  Non-uniform illumination caused by dramatic lighting effects, deep shadows, and colored glows can significantly alter image content as shown in the first row of Figure 4. Consequently, images of the same anime character may differ greatly from one another, posing a substantial challenge for computer vision and deep learning algorithms aimed at tasks such as anime character recognition.
➢ Age related appearance changes
  Time-lapse and flashbacks in the movie depict the same character at different ages, with changes in facial proportions, hairstyle, and costume introducing significant intra-class variability. A few exemplar images are shown in second row of the Figure 4.
➢ Scale changes
  Characters appear at varying distances, from extreme close-ups to small figures in wide shots, leading to the loss or exaggeration of facial details when resized to a fixed input resolution. Such variations in scale as shown in the third row of Fig. 4, introduce significant challenge for image-based anime character recognition tasks.

## Ethics Statement

The authors have read and follow the ethical requirements for publication in Data in Brief and confirming that the current work does not involve human subjects, animal experiments, or any data collected from social media platforms.

1. Terms of Service (ToS):
  The images included in this dataset were manually collected without any automated tools from publicly accessible websites that host media related to the anime *Attack on Titan -* including official promotional materials, trailers, and publicly available screenshots. We were careful to avoid scraping from any platforms that explicitly prohibit automated data collection in their Terms of Service or robots.txt files. The sources we used did not restrict this type of access at the time of collection. Still, we fully acknowledge the importance of respecting website policies and therefore limit this dataset strictly to non-commercial, academic research use.
2. Copyright:
  Attack on Titan is a copyrighted work owned by its creators and production studios (including Kodansha, Wit Studio, and MAPPA). We do not claim any ownership over the original content. The dataset contains selected still frames from the anime, which are transformed into labelled image data for non-commercial, academic research in computer vision.
  Our use follows the principles of fair use (or fair dealing), as the images are used in a new and transformative context - converted into a structured dataset for tasks like character recognition and scene analysis. This educational purpose and non-commercial intent support the justification for sharing.
  The dataset is hosted on Mendeley Data, a public academic repository, with clear mention of research-only usage. Proper attribution is provided, and all rights to the original content remain with the original copyright holders.
3. Privacy:
  This dataset contains only fictional characters and scenes from an animated series. There is no personal or private information related to real individuals. Consequently, issues of privacy and anonymization do not apply in this case.
4. Scraping policy:
  No data was collected from platforms that have specific scraping restrictions or enforce access through APIs only (such as Twitter or Netflix). Instead, the dataset was built from sources that either allow scraping under fair conditions or do not explicitly prohibit it. Our data collection was done responsibly - using rate-limiting techniques and taking care not to burden the websites’ infrastructure.

## CRediT Author Statement

**Guldasta Imraj Khan:** Conceptualization, Methodology, Dataset Collection. **Hafeez Anwar:** Supervision, Writing - review & editing. **Qasim Jan:** review and editing. **Shahid Hussain:** Corresponding author.

## Data Availability

Mendeley DataAOT Characters Dataset (Original data).
